# Is coronary artery calcium an independent risk factor for white matter hyperintensity?

**DOI:** 10.1186/s12883-023-03364-7

**Published:** 2023-08-30

**Authors:** Hui Jin, Xue Qin, Fanfan Zhao, Yuting Yan, Yu Meng, Zhenyu Shu, Xiangyang Gong

**Affiliations:** 1 Bengbu Medical College, Bengbu, 233030 China; 2 Center for Rehabilitation Medicine, Department of Radiology, Zhejiang Provincial People’s Hospital(Affiliated People’s Hospital), Hangzhou Medical College, No. 158 Shangtang Road, Hangzhou City, Zhejiang Province China

**Keywords:** Atherosclerosis, Coronary artery calcium score, White matter hyperintensity, Risk factors

## Abstract

**Background:**

Cardiovascular diseases have been considered the primary cause of disability and death worldwide. Coronary artery calcium (CAC) is an important indicator of the severity of coronary atherosclerosis. This study is aimed to investigate the relationship between CAC and white matter hyperintensity (WMH) in the context of diagnostic utility.

**Methods:**

A retrospective analysis was conducted on 342 patients with a diagnosis of WMH on magnetic resonance images (MRI) who also underwent chest computed tomography (CT) scans. WMH volumes were automatically measured using a lesion prediction algorithm. Subjects were divided into four groups based on the CAC score obtained from chest CT scans. A multilevel mixed-effects linear regression model considering conventional vascular risk factors assessed the association between total WMH volume and CAC score.

**Results:**

Overall, participants with coronary artery calcium (CAC score > 0) had larger WMH volumes than those without calcium (CAC score = 0), and WMH volumes were statistically different between the four CAC score groups, with increasing CAC scores, the volume of WMH significantly increased. In the linear regression model 1 of the high CAC score group, for every 1% increase in CAC score, the WMH volume increases by 2.96%. After including other covariates in model 2 and model 3, the β coefficient in the high CAC group remains higher than in the low and medium CAC score groups.

**Conclusion:**

In elderly adults, the presence and severity of CAC is related to an increase in WMH volume. Our findings suggest an association between two different vascular bed diseases in addition to traditional vascular risk factors, possibly indicating a comorbid mechanism.

## Introduction

White matter hyperintensity (WMH) is a prevalent brain white matter lesion that can be visualized using MRI scans. WMH can be detected in approximately 4-6% of the general population, but with the increase of age, especially in the elderly population over 60 years old, the incidence rate rises significantly [1, 2]. WMH has been considered a common diagnostic feature indicating cerebral small vessel diseases (CSVDs), and it has been closely associated with cognitive decline, major depressive disorder, Alzheimer’s disease, and stroke. Great interests have been raised in the pathological mechanisms of WMH and the associated risk factors [3–5]. Age and hypertension have been recognized as risk factors for WMH [6]. Chronic ischemia often occurs in small blood vessels in the brain, leading to demyelination and axonal loss. This has been considered the main cause of the formation of WMH [5]. Additionally, hypoperfusion resulting from hypoxia and alteration in automatic regulation of cerebral blood vessels, leakage of the blood-brain barrier, inflammation, degeneration, and cerebral amyloid angiopathy are also thought to be the causes of WMH [7]. The potential mechanisms of WMH have been widely debated, and whether WMH is influenced by systemic atherosclerosis remains unclear.

Atherosclerosis is an inflammatory disease leading to lumen stenosis and subsequent thrombosis with various complications, such as coronary atherosclerotic heart disease and stroke [8]. Coronary artery calcium (CAC) is widely considered a biomarker indicating atherosclerosis. Numerous studies have suggested that CAC can predict systemic atherosclerotic load, including cerebral and carotid arteries, besides coronary arteries [9–12]. As a common noninvasive screening method, CAC can effectively assess cardiovascular risks, which has been used for the primary prevention of cerebrovascular diseases [13, 14]. Recent studies reported that CAC score could estimate the risk of future atherosclerotic coronary artery diseases and cerebrovascular diseases [15, 16]. However, it remains to be elucidated whether CAC is an independent risk factor for WMH.

In previous studies, Suzuki et al. found that CAC may be a promising tool for predicting common subtle white matter damage, but their study was limited to middle-aged men and lacked direct correlation between CAC and WMH [17]. Choi et al. investigated the correlation between WMH and CAC in a healthy population, but the assessment of WMH was based on the Fazekas visual scale and lacked more refined measures [18]. Kim BJ et al. documented a robust association between moderate-to-extensive CAC (CAC score of ≥ 100) and increased odds of having cerebral SVDs, but the healthy volunteer effect may have introduced bias and limited generalizations of the results [2]. In this study, we aim to screen independent risk factors affecting the formation of WMH in elderly individuals through logistic regression, then we measure the volume of WMH with an automatic segmentation algorithm and construct a linear regression model to assess the effect of CAC score on WMH volume.

## Methods

All procedures and research protocols were approved by the Research Ethics Committee of Zhejiang Provincial People’s Hospital (QT2022419). Informed consent was waived by the Research Ethics Committee of Zhejiang Provincial People’s Hospital because of the use of retrospective image data. The study was conducted following the principles of the Declaration of Helsinki.

### Study population

We collected MRI data from inpatients who underwent cranial MRI and chest CT scans from March 2021 to February 2022 at our Hospital. We performed a retrospective analysis of the MRI data. The study inclusion criteria were as follows: (1) WMH could be observed on T2-weighted imaging (T2WI) and T2-fluid-attenuated inversion recovery imaging (T2-FLAIR); (2) ages were ≥ 60 years old with complete clinical information; (3) chest CT scan and brain MR were conducted within a one-month interval, and MRI examination included complete T1-weighted imaging (T1WI), T2WI, T2-FLAIR and diffusion-weighted imaging (DWI), and participants displayed no contraindications for MRI procedures; (4) no apparent stroke lesion could be detected in DWI, except for lacunar infarcts; and (5) there were no clinical signs of Alzheimer’s disease, Parkinson’s disease, multiple sclerosis, or traumatic brain damage. The exclusion criteria were as follows: (1) the existence of non-vascular originated white matter lesions, such as demyelinating autoimmune diseases, metabolism, toxicity, and infection; (2) the presence of large cerebral hemorrhage or previous large cerebral infarction with softening foci interfered with WMH classification on T2-FLAIR sequences; (3) a history of congenital heart disease, viral myocarditis, and cardiac stenting; or (4) poor image quality rated by trained specialists.

### Brain image acquisition and interpretation

Brain MRI images of all participants were obtained by two 3.0T MRI scanners (Siemens Trio 3.0 T and GE Discovery 750) using the same MR parameters and eight-channel coils. The routine examination sequences consisted of T2-weighted imaging, DWI, and T1, T2-FLAIR. As previously suggested, axial T2-FLAIR could be used to observe WMH with the following scanning parameters [19]: Repetition time/echo time = 9000/120 ms, field of view = 256 × 256 mm, matrix dimensions = 256 × 256 pixels, flip angle = 160°, echo chain = 18, bandwidth = 50, layer thickness = 5 mm, inter-slice gap = 0. The T1-FLAIR imaging was used for WMH segmentation with a repetition time = 1750 ms, echo time = 24 ms, field of view = 256 × 256 mm, resolution = 256 × 256, flip angle = 111°, echo chain = 10, bandwidth = 31.25, layer thickness = 5 mm, and interslice gap = 0.

(1) The Fazekas Visual Scale [20] was used to assess WMH on T2-FLAIR images, ranging from 0 to 3 in periventricular regions and 0 to 3 in deep regions. Based on a total score of 6, severe WMH was defined as a score ≥ 3 and a score < 3 was defined as mild WMH.

(2) All T1 and T2-FLAIR baseline images were imported into SPM12 software (https://www.fil.ion.ucl.ac.uk/spm/software/spm12/). Automated WMH segmentation was performed on the aligned T2-FLAIR images by a lesion prediction algorithm in the LST (https://www.statistical-modelling.de/lst.html) for SPM12. This was accomplished by the following steps: (1) removal of non-brain tissue, brainstem and cerebellum; (2) modification of white matter segmentation. WMH volumes were calculated in milliliters, corrected for interscan intracranial volume differences, and normalized to baseline intracranial volumes. Two experienced radiologists viewed the images independently and were blinded to the clinical data. WMH was modified manually with itk-snap software by an experienced neuroradiologist (http://www.itksnap.org/pmwiki/pmwiki.php).

### Measurement of CAC

All image data came from the Medical Center’s Picture Archiving and Communication System (PACS). Chest CT images of all participants were obtained from the same 64-layer multidetector CT scanner. CAC was defined as the number of adjacent pixels within the lesion ≥ 4 and CT density ≥ 130 HU. With a tube voltage of 120 KV, the adaptable iterative dose reduction 3D algorithm was used to adjust the effective tube current. CAC score was calculated as a sum of the weighted scores of all lesions in the four coronary vessels according to the modified Agatston score [21]. We categorized participants into 4 groups (0, 1–99, 100–399, and ≥ 400) based on the CAC scores [22].

### Covariates

Clinical and demographic information, including age, sex, diabetes, hypertension, hyperlipidemia, and heart diseases, was collected. The history of smoking and alcohol consumption was collected within the past five years. Smoking consumption was defined as ≥ 10 cigarettes per day. Alcohol consumption was defined as ≥ 40 g or ≥ 20 g of ethyl alcohol per day for men and women, respectively. Hypertension was defined as mean systolic blood pressure ≥ 140 mmHg and diastolic blood pressure ≥ 90 mmHg, or the current use of antihypertensive medication. Hyperlipidemia was defined as second-day fasting low-density lipoprotein (LDL) ≥ 130 mg/dl. Body mass index (BMI) was calculated as weight divided by squared height (kg/m^2^). Obesity was considered as BMI ≥ 25 kg/m². Heart diseases refer to the presence of ischemic heart disease or cardiac arrhythmia. Age was expressed as a continuous variable; sex was expressed as a dichotomous variable [19, 23].

### Statistical analysis

Continuous variables are presented as means ± SDs, and categorical variables are presented as frequencies and percentages. According to the data distribution, χ2 and ANOVA tests were used for comparison. χ2 test for trend was used to analyze WMH total intracranial volume and the distribution of clinical data in CAC score groups. Mild/severe WMH was chosen as the outcome variable, and a univariate logistic regression was constructed, incorporating statistically significant risk factors. A multivariate logistic regression was further constructed to identify independent risk factors for WMH.

Log-transformation was performed for non-normally distributed continuous variables, including the WMH volume and CAC score. When both the dependent and independent variables were log-transformed, the results could be estimated as a corresponding increase in the percentage of the dependent variable for every 1% increase in the independent variable. Regressions for demographic and vascular risk factors were gradually adjusted. Simple linear regression was performed for Model 1 according to the severity of CAC and the volume of WMH. Model 2 was corrected based on Model 1 plus age, sex, and intracranial volume. Model 3 was corrected based on Model 2 plus conventional vascular risk factors. All statistical analyses were performed using SPSS 25.0. A two-tailed test with a *P* value < 0.05 was considered statistically significant.

## Results

### Characteristics of participants

**Fig. 1:**
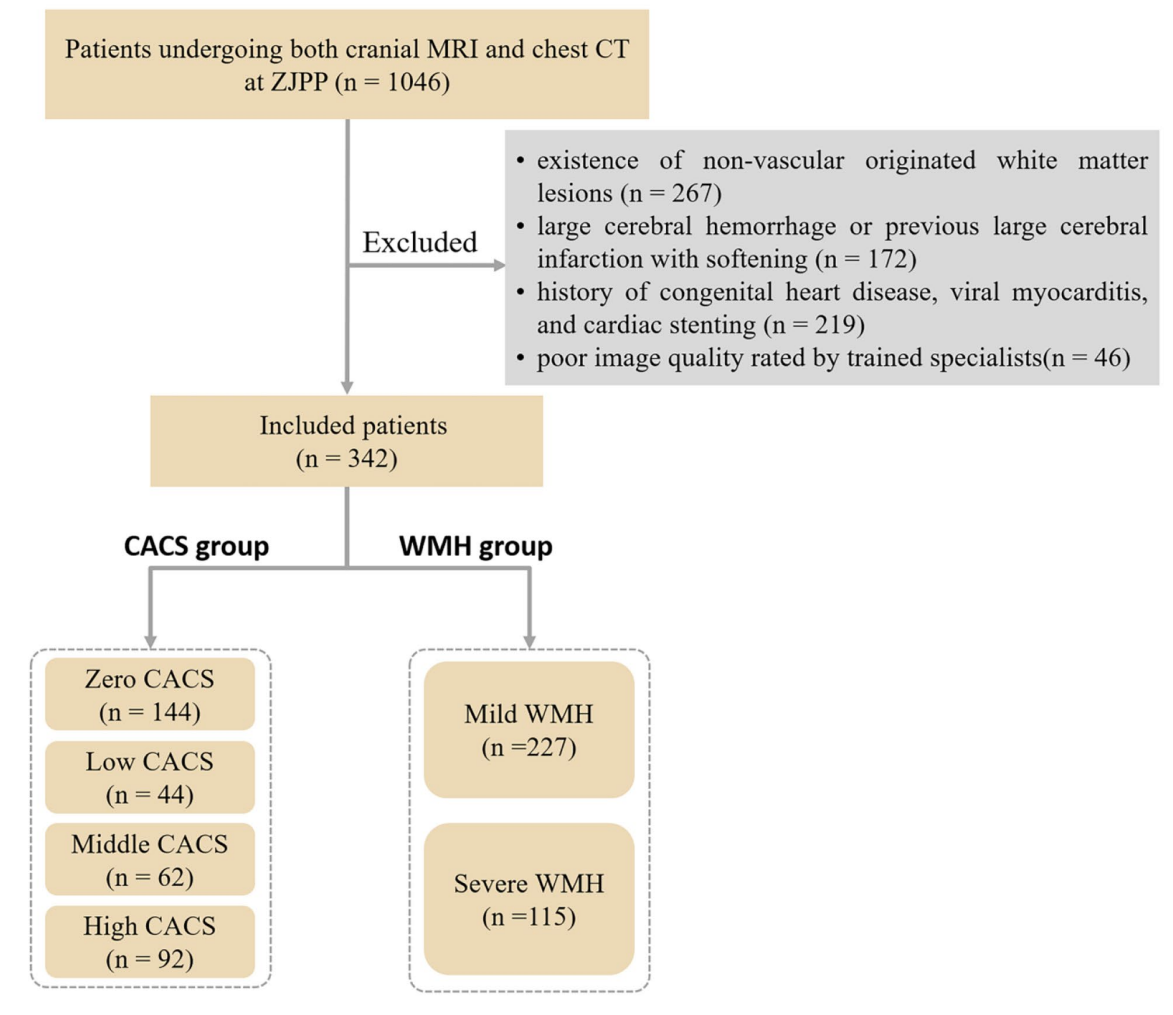
The flowchart of participant recruitment and study design. CACS, coronary artery calcium score. WMH, white matter hyperintensity

Figure 1 showed the flowchart of participant recruitment and study design. There were 704 patients excluded according to the exclusion criteria. A total of 342 (181 male, 161 female) patients between March 2021 and March 2022 were included in the analysis. Table 1 presents the demographic and clinical characteristics of patients, and Table 2 summarizes the baseline characteristics of each CAC score group. The 342 patients were classified into 4 groups based on the following criterion: zero CAC score (n = 144), low CAC score (n = 44), middle CAC score (n = 62), and high CAC score (n = 92). The age of subjects and the size of WMH volumes appear to be greater with an increase in CAC score. Additionally, the prevalence of hypertension, diabetes, heart disease, smoking and BMI had a significant positive association with CAC score. Post-hoc analysis revealed significant differences in WMH volume and age among the six paired groups (*P* < 0.001) (Zero vs. Low; b: Zero vs. Middle; c: Zero vs. High; d: Low vs. Middle; e: Low vs. High; f: Middle vs. High). Smoking and alcohol consumption were also revealed to be statistically different among the three paired groups (a: Zero vs. Low; b: Zero vs. Middle; c: Zero vs. High).

**Table 1:** Clinical and demographic information of the study sample

Mean ± SD	
Age, y	69.1 ± 8.6
Body mass index(kg/m²)	23.6 ± 3.2
Median (IQR)	
Total WMH volume, cm^3^	0.7 (0.3–4.8)
n (%)	
Sex, female	161 (47.1)
Diabetes	128 (37.4)
Smoking	104 (30.4)
Alcohol consumption	47 (13.7)
Heart disease	46 (13.5)
Hypercholesterolemia	55 (16.1)
Zero (CACS, 0)	144 (42.1)
Low (CACS, 1–99)	44 (12.9)
Middle (CACS, 100–399)	62 (18.1)
High (CACS, ≥ 400)	92 (26.9)

**Note**: WMH, white matter hyperintensity; CACS, coronary artery calcium score.

**Table 2:** Distribution of baseline characteristics in relation to the severity of CAC

Variable	Zero (CACS, 0)	Low (CACS, 1–99)	Middle (CACS, 100–399)	High (CACS, ≥ 400)		*P*		Post hoc		
n	144 (42.1)	44 (12.9)	62 (18.1)		92 (26.9)					
Age, y (mean ± SD)^*^	62.3 ± 1.6	64.1 ± 2.3	71.1 ± 2.8		80.9 ± 5.8	**< 0.001**		a, b, c, d, e, f		
Sex, female^**^	73 (50.7)	20 (45.5)	26 (41.9)		41 (44.6)	0.303				
Hypertension^**^	54 (37.5)	19 (43.2)	28 (45.2)		53 (57.6)	**0.003**		a, b		
Diabetes^**^	37 (25.7)	17 (38.6)	27 (43.5)		41 (44.6)	**0.010**		b, c, d		
Smoking^**^	19 (13.2)	17 (38.6)	26 (41.9)		42 (45.7)	**< 0.001**		a, b, c		
Alcohol consumption^**^	17 (11.8)	3 (6.8)	11 (17.7)		16 (17.3)	0.250		a, b, c		
Heart disease^**^	11 (7.6)	6 (13.6)	10 (16.1)		19 (20.7)	**0.030**		c		
Hypercholesterolemia^**^	18 (12.5)	10 (22.7)	13 (21.0)		14 (15.2)	0.050				
Body mass index (kg/m²)^*^	23.1 ± 3.3	23.4 ± 3.1	23.8 ± 2.9		24.1 ± 3.2	0.115		c		
Total WMH volume^*^	0.3 (0.2–0.4)	0.6 (0.4–0.9)	2.8 (2.2–3.8)		10.0 (6.5–14.8)	**< 0.001**		a, b, c, d, e, f		

**Note**: WMH, white matter hyperintensity; CACS, coronary artery calcium score, Data are presented as n (%), mean ± SD or median. Bold, *P* < 0.05, significantly different between groups. Post hoc analyses: a: Zero vs. Low; b: Zero vs. Intermediate; c: Zero vs. High; d: Low vs. Intermediate; e: Low vs. High; f: Intermediate vs. High. ^*^ means ANOVA test. ^**^ means chi-square test

### Association between vascular risk factors and WMH

In univariate logistic regression analysis, the outcome variable was WMH severity, age (OR 1.169; 95% CI 1.130–1.210), CAC score (OR 1.005; 95% CI 1.004–1.006), BMI (OR 1.150; 95% CI 1.066–1.240), hypertension (OR 2.435; 95% CI 1.540–3.850), diabetes mellitus (OR 2.315; 95% CI 1.455–3.684), smoking (OR 1.2.887; 95% CI 1.786–4.667), and alcohol consumption (OR 0.229; 95% CI 0.138–0.379) were associated with WMH. Hypertension and diabetes mellitus are significantly associated with WMH. However, hyperlipidemia and heart disease were not significantly associated with WMH.

**Fig. 2:**
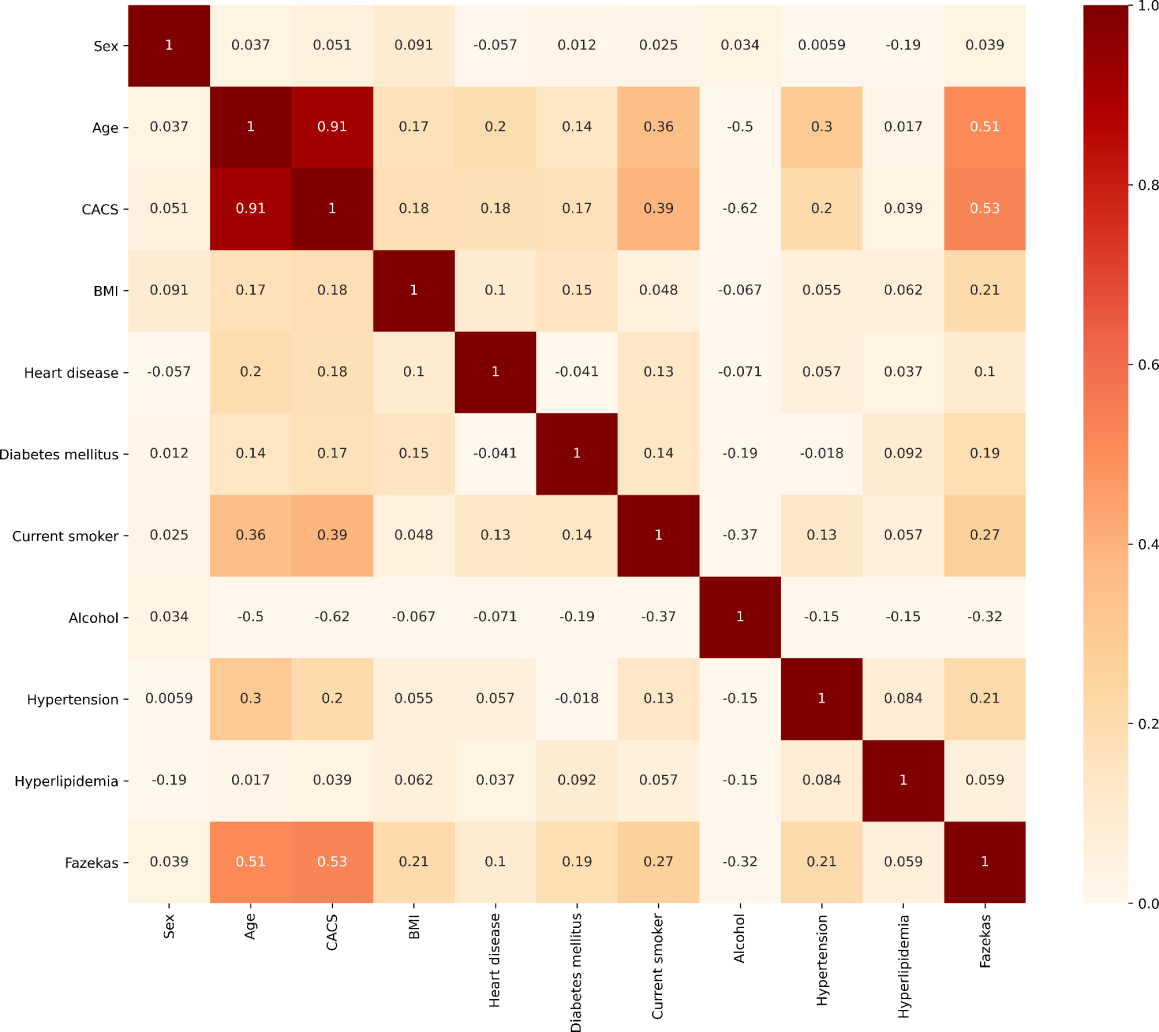
The correlation map between statistically significant clinical risk factors and white matter hyperintensity. WMH, white matter hyperintensity; CACS, coronary artery calcium score

In multivariate logistic regression analysis, hypertension (OR 1.815; 95% CI 1.042–3.162) and diabetes (OR 2.183; 95% CI 1.247–3.822) were significantly associated with WMH, but the strength of correlation was less than the previous ones. CAC score was associated with WMH, and the odds ratio was the same as in the univariate logistic regression analysis (Table 3). Figure 2 presented the correlation coefficients between WMH and the regular risk factors. CAC score and age were also positively associated with severe WMH (*r* = 0.91).

**Table 3:** Independent risk factors associated with white matter hyperintensity

	Univariate	*P* Value	Multivariate	*P* Value
	OR (95% CI)		Adjusted OR (95% CI)	
Age, y	1.169 (1.130–1.210)	**0.001**	NA	NA
Sex, male	1.180 (0.752–1.852)	0.322	NA	NA
Body mass index (kg/m²)	1.150 (1.066–1.240)	**0.001**	NA	NA
Hypertension	2.435 (1.540–3.850)	**0.001**	1.815 (1.042–3.162)	**0.035**
Diabetes	2.315 (1.455–3.684)	**0.001**	2.183 (1.247–3.822)	**0.006**
Smoking	2.887 (1.786–4.667)	**0.001**	NA	NA
Alcohol	0.229 (0.138–0.379)	**0.001**	NA	NA
Heart disease	1.805 (0.962–3.388)	0.066	NA	NA
Hypercholesterolemia	1.391 (0.768–2.517)	0.276	NA	NA
CACS	1.005 (1.004–1.006)	**0.001**	1.005 (1.004–1.006)	**0.001**

**Note**: WMH, white matter hyperintensity; CACS, coronary artery calcium score, NA, not available as the variable was not included in the multivariate logistic regression; Bold values indicate that the variables are significant with P < 0.05.

### Linear regression analyses of WMH volume and CAC score

In Model 1, for the low CAC score group, a 1% increase in Agatston score was associated with a 0.91% increase in WMH volume (95% CI, 0.83–0.99); for the moderate CAC score group, a 1% increase in Agatston score was associated with a 0.94% increase in WMH volume (95% CI, 0.90–0.97); and for the high CAC score group, a 1% increase in Agatston score was associated with a 2.96% increase in WMH volume (95% CI, 0.83–0.99). The highest-risk CAC group (≥ 400) had a greater change in WMH volume compared to the lowest-risk CAC group (< 400). Model 2 adjusted for age and sex, and while the β coefficients decreased, the significant correlation between high CAC score group and WMH volume remained. Based on Model 2, Model 3 included conventional vascular risk factors, and the *β* coefficients for all CAC score groups decreased. In model 3, for every increase of 1% in CAC score, the WMH volume of the high CAC score group increased by 0.95% [*β*, 0.95 (95% CI, 0.53–1.36)], which was still higher than the changes in WMH volume in the low and medium CAC score groups, 0.50% [*β*, 0.50 (95% CI, 0.33–0.67)] and 0.84% [*β*, 0.84 (95% CI, 0.70–0.98)] (Table 4).

**Table 4:** Multilevel mixed-effects linear regression of WMH volume and coronary artery calcium score

CACS	*β* (model 1)	95% CI	*β* (model 2)	95% CI	*β* (model 3)	95% CI
Low (1– 99)*	0.91	0.83–0.99	0.46	0.32–0.61	0.50	0.33–0.67
Middle(100–399)*	0.94	0.90–0.97	0.82	0.69–0.95	0.84	0.70–0.98
High (≥ 400)*	2.96	2.69–3.23	1.06	0.27–1.85	0.95	0.53–1.36

**Note**: WMH, white matter hyperintensity; CACS, coronary artery calcium score. *Log transformed, units are per 1 natural log unit unless otherwise noted (original units: WMH, 1 mm^3^). Model 1: Simple linear regression; Model 2: age, sex, and TIV; Model 3: model 2 + Hypercholesterolemia, diabetes (history of diabetes, fasting glucose level ≥ 126 mg/dL, or use of hypoglycemic medications), smoker (current vs. non), body mass index (kg/m²). The *P*-values for the above models are all less than 0.001

## Discussion

The results in the present study found that WMH volumes were statistically different between the four CAC score groups, with WMH volumes increased significantly with increasing CAC scores. The total WMH volume is significantly higher in the CAC high score group than in the CAC low score group and the medium WMH group. In linear model 1 of the CAC high score group, for every 1% increase in CAC score, the WMH volume increases by 2.96%. This study’s results extend the linear correlation between CAC and the volume of WMH in elderly population, which may have important implications for the early detection and prevention of cerebrovascular disease. Additionally, these relationships remained consistent after controlling for factors such as sex, age, diabetes, hypertension, hypercholesterolemia, smoking, BMI, and heart disease. Our results indicate that age may not be an independent risk factor for WMH. This result was similar to the conclusion drawn from previous pathological studies, that there was no significant correlation between age and WMH vascular density reduction [24]. Nevertheless, the majority of research results indicated that age was an independent risk factor for WMH [25]. Given our limited sample size, potential selection bias in the study population, and the strong collinearity between CAC and age, the variance inflation factor (VIF) was 19.2, The fluctuations of white matter hyperintensity may be relatively small for elderly people in this study (69.1 ± 8.6 years), which may collectively contribute to the non-significance of age in the multivariable logistic regression. Of course, we acknowledge that not being significant in multivariate regression does not mean that age is not a common independent risk factor, we need to discuss this issue with caution. Furthermore, hyperlipidemia not only causes deficits in microvascular hemodynamic regulation but also increases blood viscosity and resistance [26]. However, in the current study, hyperlipidemia appears not to be an independent risk factor for WMH, which is possible because the hyperlipidemic patients have been taking statins for a long time.

Despite the differences in clinical manifestations between heart and brain diseases, they may share similar pathophysiological mechanisms, such as atherosclerosis in coronary arteries and cerebral small vessels, because they both rely on large surface arteries to deliver blood to tissues through a network of small penetrating vessels [27]. Currently, there is great interest in identifying a biomarker that is both convenient and reliable for the assessment of both CAD and WMH. CAC is the most specific marker of subclinical atherosclerosis, the CAC score has the advantage of representing atherosclerotic burden rather than directly quantifying coronary artery calcified plaque volume. The development of CAC is closely associated with cardiovascular risk factors [28]. Recent studies have associated CAC with the development of atherosclerosis in other arteries (e.g., carotid intima-media thickness and kidney disease) [29]. CSVD represented by WMH has also been related to cardiovascular risk factors (e.g., hypertension and diabetes), and reflects the same atherosclerosis of CSVD [30]. Furthermore, the development of WMH and CAC may also associate with the impaired endothelial functional barrier in arteries [31]. When the blood-brain barrier is impaired, plasma components leak into the vessel wall and brain tissue, thus causing local inflammation and neuronal damage, local thickening of small arteries, and finally, ischemic necrosis [32, 33].

The association between WMH and CAC observed in the current study supports the idea that a common pathophysiological process (e.g., systemic atherosclerosis) may result in coronary artery and cerebral microvascular diseases. In 2021, Suzuki et al. utilized CAC as a predictor to estimate microstructural damage of the WMH in based on DTI, they observed a significant linear trend of fractional anisotropy, but not other measures, across the CAC groups after multivariable adjustment. In the secondary analyses, CAC was associated with lower fractional anisotropy in men but not in women [17]. In 2022, Choi et al. investigated the correlation between WMH and CAC in a healthy population, the results showed that categories of higher CAC scores showed increased associations with both periventricular and deep WMHs in a dose-dependent relationship, similar to most studies, they only employed the Fazekas visual scale for assessment, and poor interobserver agreement may limit the generalizability of the results [18]. This study constructed linear regression models to evaluate the effect of CAC score on WMH volume. The results showed statistically significant differences in WMH volume among the four CAC score groups, with a significant increase in WMH volume as CAC score increased. The total WMH volume in the high CAC score group was higher compared to the low and moderate CAC score groups. Moreover, in model 1 of the high CAC score group, a 1% increase in the CAC score would lead to a 2.96% increase in WMH volume. The findings of this study extend the linear correlation between CAC and WMH volume in the elderly population, which may have significant implications for the early identification and prevention of cerebrovascular diseases.

CSVD is closely related to a variety of neurological disorders, and WMH has been considered as a biomarker indicating the occurrence of cerebral small vessel diseases [5]. Increased WMH was reported to associate with executive deficits and reduced information processing speed strongly; this not only leads to cognitive decline in elderly adults [34–36], but also increases the risk of ischemic and hemorrhagic strokes [37–39]. Currently, we cannot identify the specific common mechanism of CAC and cerebral WMH or the underlying mechanisms of an asymptomatic disease process involving different vascular beds. Our focus is not on debating whether CAC or SVD are causally linked, or if CAC can predict the risk of future stroke or dementia. Such hypotheses can only be substantiated through sufficiently robust prospective studies. Instead, our aim is to identify the common pathogenic mechanisms linking these subclinical phenomena, and thus integrate cerebral SVD into the risk profile of systemic cardiovascular disease and high CAC scores.

We acknowledge the limitations of this study. Our results illustrate an association between CAC score and WMH volume, and the high-risk CAC score group has a greater effect on WMH volume. This indicates a common pathophysiological process between WMH and coronary artery calcium (systemic atherosclerosis). To be specific, we acknowledge that this is a retrospective study, results from which cannot explain a causal relationship. Longitudinal exposure to risk factors, as well as the relative severity, were not taken into consideration, thus increasing the possibility that unknown confounding factors may have contributed to the greater proportion of WMH and CAC in our patients. Patients in the current study were elderly adults, they appeared to have more exposure to vascular risk factors. The patients without WMH were excluded in this study, it seems there may be a selection bias in some extent. However, the focus of this study is on the relationship between visible WMH and CAC score in the elderly population. For patients with subtle white matter injury, we are temporarily unable to include and accurately analyze the association with CAC in the absence of visualization. Additionally, we used an automatic segmentation algorithm to extract WMH volume to explore the linear correlation between WMH volume and CAC score. Therefore, we included the elderly population with visible WMH in T2WI and T2-FLAIR. The results of this study cannot be generalized to the general population. Given the small scale of this study, we did not take the progression of WMH into consideration. Therefore, long-term prospective studies are still required at a later stage.

In conclusion, this study demonstrates that the CAC score in the elderly population was linearly correlated with WMH volume, and individuals in the high-risk CAC score group have a higher WMH volume. In the future, we may be able to identify people at increased risk for catastrophic vascular events by deploying a CAC score to achieve early detection and intervention of WMH.

## Data Availability

The datasets generated and analyzed during the current study are not publicly available due to patient privacy concerns but are available from the corresponding author on reasonable request.

## Abbreviations

CAC: Coronary artery calcium
CACS: Coronary artery calcium score
WMH: White matter hyperintensity
CSVD: Cerebral small vessel disease
CT: Computed tomography
MRI: Magnetic resonance imaging
T2WI: T2-weighted imaging
T1-FLAIR: T1-Fluid attenuated inversion recovery
T2-FLAIR: T2-Fluid attenuated inversion recovery
DWI: Diffusion-weighted imaging
IQR: Interquartile range
OR: Odds ratio
CI: Confidence interval
